# Habitual physical activity modulates cardiometabolic health in long-term testicular cancer survivors

**DOI:** 10.1007/s00520-023-08000-1

**Published:** 2023-08-26

**Authors:** Ali Amiri, Patrik Krumpolec, Michal Mego, Barbara Ukropcová, Michal Chovanec, Jozef Ukropec

**Affiliations:** 1 Department of Metabolic Disease Research & Center of Physical Activity Research, Institute of Experimental Endocrinology, Biomedical Research Center, Slovak Academy of Sciences, Bratislava, Slovakia; 2 2nd Department of Oncology, Faculty of Medicine, Comenius University and National Cancer Institute, Bratislava, Slovakia; 3 Institute of Pathophysiology, Faculty of Medicine, Comenius University, Bratislava, Slovakia

**Keywords:** Testicular germ cell tumor, Habitual physical activity, Cardiometabolic health, Metabolic syndrome, Cisplatin-based chemotherapy

## Abstract

**Purposes:**

Physical activity (PA) may mitigate late cardiometabolic toxicity of cisplatin-based chemotherapy in testicular germ cell tumor (TGCT) long-term survivors. In this cross-sectional study, we evaluated the effects of habitual PA on metabolic syndrome (MetS) prevalence, and on the markers of cardiometabolic health and chronic inflammation in a population of long-term TGCT survivors.

**Methods:**

MetS prevalence was evaluated, and habitual PA was assessed using Baecke’s habitual PA questionnaire in TGCT survivors (*n*=195, age=41.1±8.1years, 11.7±5.2years post-therapy) and healthy male controls (*n*=41, age=38.2±8.8years). Participants were stratified into low- and high-PA groups based on median values. Differences were examined between low- and high-PA groups (in the entire sample, TGCT survivor sub-samples differing in disease stage, and healthy controls), and between TGCT survivors and controls. Next, TGCT survivors were stratified into age- and BMI-matched sub-groups based on post-treatment time (5–15/15/30years) and number of chemotherapy cycles (≤3/>3), allowing us to detect age- and BMI-independent effects of habitual PA on cardiometabolic health in the given TGCT survivor sub-populations. A correlation matrix of habitual PA and sport activity with cardiometabolic and pro-inflammatory markers was generated.

**Results:**

TGCT survivors had higher MetS prevalence than controls. Patients with high habitual PA had lower waist circumference and Systemic Inflammation Index. Habitual PA scores correlated positively with HDL-cholesterol and negatively with waist circumference and atherogenic risk. Furthermore, cardiometabolic benefits of habitual PA were more pronounced in patients with disease stages 1 and 2. Effects of habitual PA on patients sub-populations stratified by chemotherapy dose and post-treatment time clearly showed that higher levels of habitual PA were associated with lower numbers of MetS components, except for patients who received more than 3 chemotherapy cycles and were examined more than15 years post-therapy.

**Conclusions:**

Higher levels of habitual PA effectively mitigated cardiometabolic toxicity in TGCT survivors. Patients with higher cumulative doses of chemotherapy may need structured exercise interventions involving higher-intensity physical activity to achieve significant improvements in cardiometabolic health.

**Supplementary Information:**

The online version contains supplementary material available at 10.1007/s00520-023-08000-1.

## Introduction

Testicular germ cell tumors (TGCT) have been a perfect example for transforming once fatal disease into a curable one with a 10-year survival rate of more than 95%, mostly due to high sensitivity to chemotherapy [1, 2]. TGCT and its treatments often lead to a range of acute and late toxicities, promoting cardiovascular and metabolic diseases, cognitive impairment, cancer-related fatigue, hypogonadism, secondary cancers, anxiety, depression, and peripheral neuropathy [3–6]. A comprehensive examination of the cumulative burden of morbidity was examined in more than 1200 TGCT survivors who were at least 1-year after therapy. Approximately 19% of the TGCT survivors had high cumulative morbidity score, 30% medium, about 46% low or very low, and only about 5% were without any morbidities. The number of platinum-based chemotherapy cycles (≥4), smoking, and older age increased the risk of high cumulative morbidity score by 44%, 28%, and 18%, respectively. On the contrary, patients reporting regular intensive exercise (≥6 METh/week, 68.8% of the population) had 32% lower risk of high cumulative morbidity score [7]. Epidemiological data suggests a twofold risk increase in developing cardiovascular disease during 10 years of follow-up in TGCT survivors [8]. Adverse effects arising from TGCT and/or its treatment are notably influencing patients’ quality of life and are considered an important survivorship dilemma for this population. Preliminary evidence indicates that regular exercise has the capacity to mitigate the risk [9].

Level of systemic inflammation, a hallmark of cancer pathophysiology, could be defined by the systemic immune inflammation index (SII) [10]. Capacity of SII in predicting tumor progression and survival rate in TGCT was demonstrated [11]; however, the impacts of habitual physical activity, regular exercise, or physical fitness on SII and its interrelations with late cardiometabolic health have not yet been evaluated in TGCT survivors.

Growing body of evidence indicates that regular physical activity brings benefits to patients with testicular germ cell tumor and that safely guided regular exercise training is an effective strategy to maintain or improve physical fitness and quality of life during and after treatment [9, 12]. Recent systematic review clearly showed that 139 out of 140 independent meta-analyses on clinical trials evaluating the effects of exercise on cancer patients and survivors showed beneficial impact of exercise on psychosocial, physical, and functional outcomes. Moderate effect sizes were found for cardiovascular fitness and muscle strength and small for cancer-related fatigue, health-related quality of life, and depression [13]. Physical activity guidelines for cancer patients and survivors exist and we do advocate for their implementation into clinical practice whenever possible [14–16]. The American College of Sports Medicine (ACSM) guidelines emphasize that exercise training is generally safe for cancer survivors, and that survivors should “avoid inactivity.” Provided evidence allowed to conclude that 75 min of high-intensity or 150 min of moderate-intensity aerobic activities per week, 2–3 sessions of the resistance training targeting the large muscle groups per week, and/or their combination improve common cancer-related health outcomes, including anxiety, depressive symptoms, fatigue, physical functioning, and health-related quality of life. Implications for the peripheral neuropathy and cognitive functioning remained uncertain [16, 17]. Many cancer patients remain inactive during and after successful treatment. It is true that implementation of a safe and effective individualized exercise regimen for TGCT patients during chemotherapy could be challenging as it requires effective cooperation of health care professionals dedicated to deliver and closely monitor effects of both cancer therapy and exercise [18, 19]. It is, however, important to note that exercise intervention programs in cancer patients are well tolerated, if professional supervision is provided to ensure efficacy and safety [12, 20].

In this work, we examined the association between the habitual physical activity and cardiometabolic health in TGCT survivors while considering the cancer stage, chemotherapy dose, and post-treatment time in age- and BMI-matched patient groups.

## Materials and methods

### Patient population

Study population included 195 TGCT survivors (age: 41.1±8.1 years, >5years post-therapy, BMI: 27.1±3.7 kg/m^2^) who underwent cisplatin-based chemotherapy (BEP; bleomycin, etoposide, cis-platinum) and 41 healthy age and BMI matched male individuals (age: 38.2±8.8 years; BMI: 27.0±3.8 kg/m^2^) examined between 2017 and 2021 in whom habitual physical activity (PA) was evaluated with a validated questionnaire [21]. To determine the effects of habitual physical activity, patients were stratified into high- and low-PA groups based on median value of habitual physical activity index in the entire study population (Table 1). Patient population was next stratified according to the disease stage and ninety-eight TGCT survivors were stratified into 3 age- and BMI-matched groups differing by the number of chemotherapy cycles (dose) and post-treatment time: group A — patients with ≤ 3 cycles of chemotherapy examined 5–15 years post-therapy (*n*=39); group B — patients with > 3 cycles of chemotherapy examined 5–15 years post-therapy (*n*=39); and group C — patients with > 3 cycles of chemotherapy examined 15–30 years post-therapy (*n*=20). Patients with ≤ 3 cycles of chemotherapy examined 15–30 years post-therapy were very rare in our cohort and therefore could not be examined and compared to 41 healthy age- and BMI-matched controls recruited by social media from the general population (Table 3). None of the examined population of TGCT survivors received radiotherapy.

**Table 1:** Characteristics of the study population based on physical activity

Parameter	Entire TGCT cohort	Low physical activity group	High physical activity group
Number of patients	195	91	104
Age (yrs.)	41.1±8.1 (195)	41.7±8.0	40.4±8.1
Age at CT (yrs.)	29.4±7.9 (161)	30.5±8.0	28.2±7.8
Years after CT (yrs.)	11.7±5.2 (161)	11.8±5.8	11.6±4.7
No. of CT cycles	3.3±1.7 (151)	3.3±1.6	3.3±1.7
Stage 1 disease (A, B, S)	61 (23, 27, 11)	32 (14, 15, 3)	29 (9, 12, 8)
Stage 2 disease (A, B, C)	56 (16, 28, 12)	18 (4, 9, 5)	38 (12, 19, 7)
Stage 3 disease (A, B, C)	77 (21, 18, 38)	41 (11, 8, 22)	36 (10, 10, 16)
BMI (kg m^−2^)	27.1±3.7 (169)	27.5±3.9	26.8±3.6
Total cholesterol (mmol/L)	5.47±1.00 (191)	5.60±1.08 (89)	5.34±0.92 (102)
LDL-cholesterol (mmol/L)	3.21±0.91 (191)	3.34±0.99 (89)	3.09±0.81 (102)
Systemic Immune Infl. Index	518.4±259.0 (98)	**564.5±299.2** (47)	466.3±195.7* (51)
Testosterone (ng/mL)	6.28±3.99 (164)	5.69±3.25 (75)	6.84±4.54 (89)
Sport index	2.69±0.79 (195)	**2.22±0.59** (91)	3.15±0.67** (104)
Total habitual PA	8.22±1.29 (195)	**7.47±0.74** (91)	9.57±0.78** (104)
Waist circumference (cm)	98.4±9.2 (176)	**100.2±8.8** (81)	96.7±9.2* (95)
Waist circumference ≥102 cm	64/176 (36.4%)	36/81 (44.4%)	28/95 (29.4%)
BP systolic (mmHg)	138.3±17.3 (179)	137.7±14.3	138.9±19.8
BP diastolic (mmHg)	87.8±11.6 (179)	88.1±11.1 (84)	87.4±12.0 (95)
BP sys/dia ≥ 130/85 mm	119/179 (66.5%)	55/84 (65.5%)	64/95 (67.4%)
HDL-cholesterol (mmol/L)	1.44±0.35 (191)	1.40±0.33 (89)	1.48±0.37 (102)
HDL-cholesterol <1.03 mM	17/191 (8.9%)	7/89 (7.9%)	10/102 (9.8%)
Triglycerides (mmol/L)	1.80±1.07 (191)	1.96±1.15 (89)	1.67±0.98 (102)
Triglycerides ≥1.7mM	85/191 (44.5%)	47/89 (52.8%)	38/102 (37.3%)
Fasting glucose (mmol/L)	5.60±1.03 (194)	5.62±1.15 (90)	5.58±0.92 (104)
Fasting glucose > 5.6 mM	72/194 (37.1%)	34/90 (37.8%)	38/104 (26.5%)
Number of MetS components	1.93±1.30 (195)	2.09±1.22 (91)	1.79±1.36 (104)
None MetS component	13.8%	12.1%	15.4%
One MetS component	29.7%	19.8%	38.5%
Two MetS components	20.5%	27.5%	14.4%
Three MetS components	24.1%	29.7%	19.2%
Four MetS components	9.2%	9.9%	8.6%
Five MetS components	2.6%	1.1%	3.8%

Significant differences between low and high physical activity groups are marked by bold

Data are presented as mean ± STDV, *p<0.05, **p<0.01

Number of observations in parentheses; *CT*, chemotherapy; *BMI*, body mass index; *BP*, blood pressure; *ALT*, alanine aminotransferase; *AST*, aspartate aminotransferase; *Systemic Immune Infl. Index.*, systemic immune inflammation index; *PA*, physical activity; *MetS*, metabolic syndrome.

### Patients’ recruitment and examination

Patients were recruited and screened for eligibility at the outpatient clinic of the National Oncology Institute, and at the Research clinic of the Biomedical Research Center SAS, Bratislava, Slovakia (2017–2021). The study flowchart illustrates patient recruitment and stratification strategies (Supplementary Fig. 2). Physician/trained nurse collected the following data: habitual physical activity scores (Beacke’s habitual physical activity questionnaire), anthropometric data (body weight and height, waist circumference measured in the mid-point between the lowest rib and the iliac crest), blood pressure (OMRON HEM-907, Japan), and a fasting blood sample to measure lipid profile (triglycerides, total, HDL- and LDL-cholesterol, glycemia, blood count, ALT and AST activity, and testosterone). Systemic immune inflammation index was calculated using formula SII=(*P*×*N*/*L*), where *P*, *N*, and *L* refer to counts of circulating platelets, neutrophils, and lymphocytes, respectively [11]. Atherogenic risk index was calculated as total-cholesterol to high-density lipoprotein (HDL) cholesterol ratio [22]. Metabolic syndrome (MetS) was defined according to the American Heart Association/National Heart, Lung, and Blood Institute (AHA/NHLBI) [23] as the presence of ≥3 of the following components: serum triglycerides ≥1.7 mmol/L or being under lipid-lowering treatment; fasting serum glucose > 5.6 mmol/L or being under glucose-lowering treatment; blood pressure (a systolic blood pressure ≥130 mm Hg, diastolic blood pressure ≥85 mm Hg, or being under anti-hypertensive treatment); HDL-cholesterol <1.03 mmol/L; and waist circumference ≥102 cm.

Study protocol was approved by the Ethics Committee of the National Cancer Institute in Bratislava and conforms to ethical guidelines of the Helsinki declaration of 1964 (2013 revision). All participating individuals signed a written informed consent prior entering the study.

### Habitual physical activity assessment

Baecke’s Habitual Physical Activity Questionnaire [11, 21] was developed to measure self-reported levels of habitual physical activity behavior during the last 12 months considering five domains of physical activity (leisure-time, sports practice, occupational, transportation, and household). The principal components analysis revealed three conceptually meaningful factors from a total of sixteen items. These factors were interpreted as follows: (1) physical activity at work; (2) sport during leisure time; and (3) physical activity during leisure time excluding sport. This questionnaire can be used repeatedly to assess changes in habitual physical activity as it covers qualitative and quantitative indexes and addresses a variety of physical activity dimensions. Validity, reproducibility, and reliability were tested in various populations [24–26] including cancer survivors [27, 28].

Since the questionnaire scores have no inherent unit of measure (e.g., kcal/unit time), scoring system was designed to represent a general classification of activity based on the proportion of a year during which the individual performs physical activity (frequency), intensity, and time (duration) for all the (i) work-related, (ii) sport, and (iii) non-sports-leisure time physical activities. The overall habitual physical activity score is the sum of the three components. As such, the habitual sport activity index represents patient’s involvement (frequency, duration, intensity) in sport-related activities, i.e., structured exercise activities, while the total PA score encompasses a wider range of activities, including occupational and leisure-time non-exercise physical activities, including household activities.

Strong association between the Beacke’s habitual physical activity questionnaire scores and objective habitual physical activity measurements obtained over a 7-day period using accelerometers (GeneActive, Activinisghts, UK) confirmed a reliability (validity) of our stratification strategy in an independent TGCT survivors’ cohort (*n*=38, age 42±8 years, BMI 26.9±4.0 kg m^−2^, post-treatment time 8.0±7.0 years), which is a part of the ongoing exercise-intervention study (Supplementary Fig. 1).

### Statistical analysis

A priori power analysis (G*Power, Germany) was based on differences in waist circumference related to high and low habitual physical activity index in a healthy men population of similar age from our published study [29]. We worked with the assumption that similar group effect could be expected in the healthy and the TGCT population, set the probability of type I error (*α*) to 0.05 and power (1 − *β*) to 0.95. Power analysis showed that in the population/sample, size of 26/13 would be sufficient to reveal significant and physiologically relevant difference. Further statistical analyses were performed using SAS Jump (USA). Data were tested for normal distribution. Unpaired *t*-test and general linear model were used to assess differences between the two experimental groups (low vs high PA). Patients were stratified into low and high habitual PA groups according to the median value of the overall habitual PA in the entire population.

More than two group differences were evaluated by one-way ANOVA with the Tukey-Kramer post hoc test. Results are given as means ± SD. Spearman correlation analysis was performed to generate correlation matrix of a total physical activity and sport activity indices with metabolic syndrome components, cardiometabolic risk, and pro-inflammatory risk parameters (SII — systemic immune inflammation index) [11]. The correlation matrix corresponding to the entire study population could be directly compared with that found in sub-populations defined by the disease stage and in age-matched healthy controls. Stepwise multiple regression analysis was used to test if any of these parameters (habitual PA, disease stage, chemotherapy dose (number of cycles), and post-treatment time) are age and BMI independent determinants of metabolic syndrome (MetS components: waist circumference, blood pressure, HDL-cholesterol, triglycerides, fasting plasma glucose). Statistical significance was considered low at ^*^p < 0.05; moderate at ^**^p < 0.01; strong at ^***^p < 0.001; and trend ^#^*p* < 0.10.

## Results

Level of habitual physical activity (PA) was determined in 195 TGCT survivors who were examined 11.7±5.2 years post-therapy and are characterized in detail in Table 1. Median value of habitual physical activity index 8.5 (IQR 1.82) was used to stratify patients in groups of high and low habitual PA. Higher levels of physical activity and sport activity in patients’ everyday life were associated with consistently lower waist circumference (− 3.6%), even more was pronounced the activity-related difference in systemic immune inflammation index (− 21.0%) and lower was also the total number of MetS components (Table 1). Diagnostic criterium for metabolic syndrome (≥3 MS components) was fulfilled in 35.9% of the whole TGCT survivors’ population, 40.7% of patients in low, and 31.6% of patients in high PA group. Waist circumference, fasting triglycerides, and glucose were the most affected MetS components when considering their diagnostic cut-off value; however, in absolute numbers, waist circumference was the only PA-modulated parameter (Table 1). Both habitual PA and its sub-category habitual sport activity (the component of habitual physical activity providing higher intensity PA) correlated negatively with waist circumference, fasting triglycerides, BMI, and with the atherogenic risk index (Table 2.) in an entire study population. Correlations with the other MetS components were less pronounced (Table 2.). Multiple regression analysis was performed to evaluate individual contribution of habitual physical activity, disease stage, chemotherapy dose (number of chemotherapy cycles), and years from chemotherapy to abdominal adiposity (waist circumference, the only MetS component regulated by habitual physical activity in this cohort). It revealed that the level of habitual PA was the best predictor of the age-adjusted waist circumference in this patient population (*R*^2^ = 0.083, *F*-ratio = 9.67, *p* = 0.0025), while the number of chemotherapy cycles (*F*-ratio = 0.921, *p* = 0.3395), years from chemotherapy (*F*-ratio 1.297, *p* = 0.2579), and disease stage (*F*-ratio = 2.022, *p* = 0.1580) did not predict abdominal adiposity.

**Table 2.**
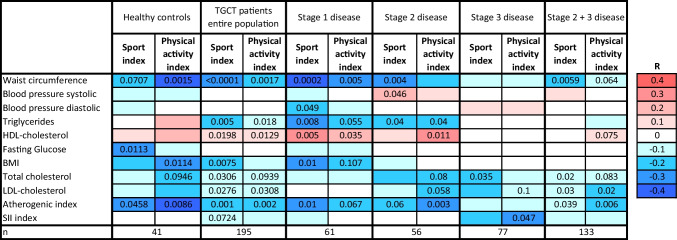
The correlation matrix of physical activity and sport activity index with metabolic syndrome components and cardiometabolic, proatherogenic, and proinflammatory risk parameters

Correlation analysis was performed in 195 TGCT survivors and 41 healthy controls. *p*-values corresponding to significant correlations are shown within the matrix. Color coding represents *R*-values of Spearman’s correlation coefficient for each correlation (red for positive and blue for negative correlations). *TGCT*, testicular germ cell tumors; *CT*, chemotherapy; *SII*, systemic immune inflammation index; *BMI*, body mass index

**Fig. 1:**
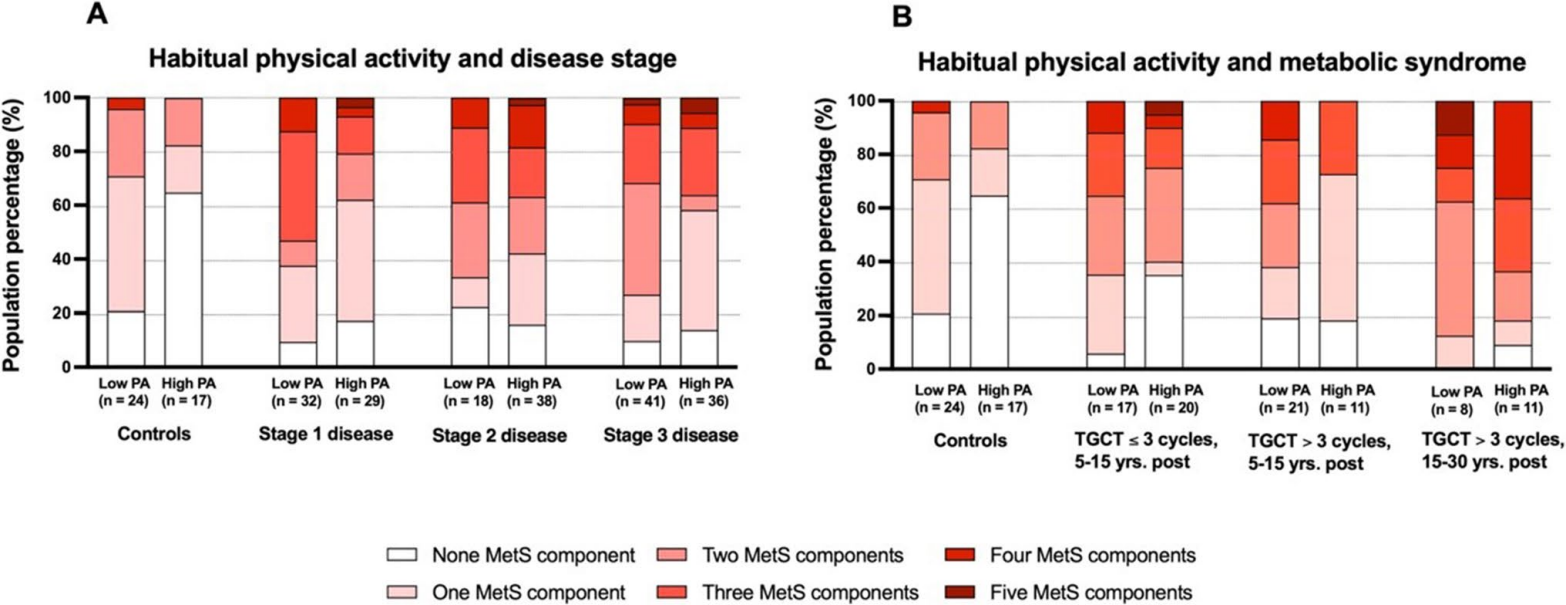
Number of metabolic syndrome components is specifically modulated by habitual physical activity in 194 TGCT survivors with different disease stage (**A**) and in 88 age- and BMI-matched patients stratified according to the chemotherapy dose (<3 vs. >3 cycles) and post-treatment time (**B**). PA, physical activity; TGCT, testicular germ cell tumors; MetS, metabolic syndrome

Stratification according to the disease stage showed that higher habitual physical activity is associated with a lower number of MetS components most significantly in patients with the stage 1 of the disease (Fig. 1A). When examined in more detail, habitual physical activity and sport activity index correlated negatively with BMI, abdominal adiposity, and atherogenic risk index in healthy control population as well as in the entire TGCT survivors’ population examined on average 11.7±5.2 years post-chemotherapy (3.3±1.7 cycles). The similar correlation pattern with specific differences was found in controls and stage 1 and 2 TGCT survivors. However, correlation pattern was different in the patient population with stage 3 (Table 2.), indicating that habitual physical activity and/or sport activity brings objectively measurable benefits mitigating cardiometabolic health specifically in stage 1 and 2 cancer survivors.

With respect to chemotherapy dose, we performed analysis of TGCT survivors stratified according to the number of chemotherapy cycles (≤3 cycles vs. >3 cycles) and aimed to examine this in relation to post-treatment time (5–15 years (median 10 years, IQR 5) vs. 15–30 years (median 18 years, QR 3) post-treatment) (Table 3). It is important to note that here we compared patient populations matched for age, BMI, and waist circumference, and that comparable healthy control man population has also been examined. There were no obvious differences in the level of habitual physical activity or sport activity between the cancer survivor groups and between survivors and healthy controls (Table 3). However, stratification of individual patients’ sub-population and control population to low and high PA sub-groups clearly showed that the sub-populations with high PA index always had lower prevalence of MetS components than their counterparts with low level of habitual PA (Fig. 1B).

**Table 3:** Characteristics of the study population based on treatment received

Parameter	Healthy controls	≤3 CT cycles 5–15 yrs. post-CHT	>3 CT cycles 5–15 yrs. post-CHT	>3 CT cycles 15–30 yrs. post-CHT
Group	CON	A	B	C
Number of patients	41	39	39	20
Age (yrs.)	38.2±8.8	40.1±7.2	41.2±7.8	42.0±6.7
Age at CT (yrs.)	-	30.0±7.6	30.3±8.2	23.2±5.6
Years after CT (yrs.)	-	10.1±3.4	11.0±3.7	18.8±3.8
Number of CT cycles	-	2.7±0.4	4.3±0.6	4.7±1.6
BMI (kg m^−2^)	27.0±3.8	26.9±3.8	27.2±3.8	28.7±4.4
Total cholesterol (mmol/L)	4.48±0.9^***A,B,C^	5.62±0.8^***CON^	5.35±1.0^***CON^	5.81±1.48^***CON^
LDL-cholesterol (mmol/L)	2.57±0.6^***A,B,**C^	3.33±0.8^***CON^	3.24±0.9^***CON^	3.36±1.37^**CON^
Atherogenic index	3.48±0.7^**A,B, ***C^	4.08±1.1^**CON,*C^	4.05±1.1^**CON,*C^	4.93±1.68^***CON,*A,B^
ALT (ukat/L)	0.21±0.13^***A,B,C^	0.69±0.30^***CON^	0.66±0.27^***CON^	0.80±0.42^***CON^
AST (ukat/L)	0.31±0.11^#A,*B,***C^	0.36±0.12^#CON,**C^	0.39±0.16^*CON, #C^	0.49±0.25^***CON,**A, #B^
Systemic Immune Infl. Index	-	440.1±142.2^#B^	582.5±328.1^#A^	592.4±441.4
Testosterone (ng/mL)	-	5.21±3.25	6.41±4.10	5.84±2.96
Sport index	2.60±0.8	2.63±0.7	2.37±0.7^#C^	2.75±0.70^#B^
Total habitual physical activity	8.16±1.4^#C^	8.65±1.5	8.21±1.2^#C^	8.80±1.02^#CON,B^
Waist circumference (cm)	97.4±10.8	97.7±9.5	100.0±10.0	101.1±12.4
Waist circumference ≥102 cm	15/41 (36.6%)	11/39 (28.2%)	14/39 (35.9%)	9/20 (45.0%)
BP systolic (mmHg)	126.8±15.7^*A,**B,C^	134.8±16.5^*CON^	138.8±16.4^**CON^	138.1±11.8^**CON^
BP diastolic (mmHg)	75.8±9.3^***A,B,C^	86.7±11.2^***CON^	87.8±11.7^***CON^	89.8±10.6^***CON^
BP sys/dia ≥ 130/85 mm	11/41 (26.8%)	26/39 (66.6%)	28/39 (71.8%)	17/20 (85.0%)
HDL-cholesterol (mmol/L)	1.31±0.3^*A^	1.46±0.4^*CON,#C^	1.38±0.3	1.27±0.41^#A^
HDL-cholesterol <1.03 mM	2/41 (4.9%)	3/39 (7.7%)	3/39 (7.7%)	5/20 (25.0%)
Triglycerides (mmol/L)	1.28±0.7^**A,*B,***C^	1.84±0.9^**CON,*C^	1.60±0.7^*CON,**C^	2.45±1.24^***CON,*A,**B^
Triglycerides ≥1.7mM	9/41 (21.9%)	20/39 (51.3%)	16/39 (41.0%)	14/20 (70.0%)
Fasting glucose (mmol/L)	4.62±0.4^***A,B,C^	5.47±0.7^***CON^	5.42±0.6^***CON^	5.61±0.95^***CON^
Fasting glucose > 5.6 mM	0/41 (0.0%)	12/39 (31.6%)	13/39 (33.3%)	8/20 (40,0%)
Fasting glucose > 7.0 mM	0/41 (0.0%)	1/39 (0.0%)	0/39 (0.0%)	1/20 (5,0%)
Number of MetS components	0.90±0.9^***A,B,C^	1.85±1.3^***CON,*C^	1.85±1.3^***CON,*C^	2.65±1.27^***CON,*A,B^
None MetS component	16/41 (39.0%)	8/39 (20.5%)	6/39 (15.4%)	1/20 (5.0%)
One MetS component	15/41 (36.6%)	6/39 (15.4%)	11/39 (28.2%)	2/20 (10.0%)
Two MetS components	9/41 (21.9%)	14/39 (35.9%)	10/39 (25.6%)	7/20 (35.0%)
Three MetS components	0/41 (0.0%)	7/39 (17.9%)	8/39 (20.5%)	4/20 (20.0%)
Four MetS components	1/41 (2.4%)	3/39 (7.7%)	3/39 (7.7%)	5/20 (25.0%)
Five MetS components	0/41 (0.0%)	1/39 (2.6%)	1/39 (2.6%)	1/20 (5.0%)

*CT*, chemotherapy; *BMI*, body mass index; *BP*, blood pressure; *ALT*, alanine aminotransferase; *AST*, aspartate aminotransferase; *Systemic Immune Infl. Index*, systemic immune-inflammation index; *MetS*, metabolic syndrome. Metabolic syndrome is diagnosed in individuals with ≥3 MetS components

Data are presented as mean ± STDV, ^*^p<0.05, ^**^p<0.01, ^***^p<0.001, ^#^*p*<0.10

## Discussion

Metabolic syndrome in present study was found in 35.9% of TGCT survivors with the median age of 41.1 years. Stratification of patients according to the level of habitual physical activity (PA) showed that those with higher level of habitual PA had lower number of MetS components, lower waist circumference, higher HDL-cholesterol, and lower systemic immune inflammation index, pointing at better cardiometabolic health (lower cardiometabolic risk) in patients with high habitual PA score (Table 1). Evidence suggests that regular exercise can improve whole-body cardiometabolic health by reducing chronic inflammation as well as improving insulin sensitivity, glucose metabolism, lipid oxidation, and metabolic flexibility [30]. Compared to 28 non-exercising TGCT patients, a short-term 12-week supervised high intensity interval training (HIIT) was effective in improving overall cardiovascular diseases (CVD) risk in 35 TGCT survivors with a median of 8 years after diagnosis who received surgery, radiotherapy, or chemotherapy [31]. Improvements included cardiorespiratory fitness (VO2max), low-density lipoproteins, high-sensitivity C-reactive protein, arterial stiffness, and carotid artery intima-media thickness. While levels of total cholesterol, HDL-cholesterol, and triglycerides were not modulated by exercise intervention, the Framingham’s risk score (FRS) was significantly reduced [31].

Our results indicate that higher habitual PA is paralleled by lower BMI, abdominal adiposity, and reduced atherogenic risk in both healthy controls and in the TGCT survivors. Higher habitual PA levels were linked to the more favorable circulating lipid profile, exemplified as lower triglycerides, total and LDL-cholesterol, and increased HDL-cholesterol, in the entire population of TGCT survivors. Correlations between habitual physical activity score and cardiometabolic parameters were found in stage 1 and 2 TGCT survivors and in healthy controls; however, a distinct correlation pattern was found in those with stage 3 disease (Table 2.), suggesting lower impact/less health benefits of higher habitual physical activity in the patients with more severe disease. It is plausible to speculate that a long-term structured exercise intervention program employing moderate to vigorous activities would be necessary to bring detectable cardiometabolic benefits to stage 3 TGCT survivors. Van Roekel et al. recently reported that moderate to vigorous physical activity is required to observe changes (between 6 weeks and 24 months post-treatment), i.e., time-related intra-individual associations between physical activity level and plasma metabolites in I–III colorectal cancer patients [32]. It is important to note that further mechanistic studies are needed to show whether changes in these metabolites may affect prognosis, but low-intensity activity was not effective in eliciting the change.

Level of systemic immune inflammation index (SII) was negatively correlated with the level of physical activity in patients of stage 3 disease. Previous studies showed that high SII was linked to poor prognosis in several malignancies such as TGCT [11], breast cancer [33], pancreatic cancer [34], and colorectal cancer [35]. The level of SII was significantly higher in 4828 people who died due to CVD, cancer, and other causes in a study of middle-aged and older general population (*n*=25761), with a median follow-up of 7.6 years. Physical activity was negatively, and BMI and smoking positively associated with SII levels [36]. To our knowledge, there is no study focusing on the relationships and/or effects of physical activity on SII in adult cancer patients. With the respect to confirmed beneficial effects of regular physical activity for cancer survivors, our observation of higher SII index in survivors with low physical activity levels might mirror poor cardiorespiratory fitness associated with more frequent occurrence of comorbidities [37, 38].

The fact that there were no obvious differences in the level of habitual physical activity between cancer survivors and healthy controls in our study is in line with results by Haugnes et al. who studied 1135 TGCT patients treated with surgery, radiotherapy, or chemotherapy (median age 43 years, median of time since diagnosis 11.1 years) and 1150 healthy control men (median age 48 years) and observed no differences in the level of physical activity between TGCT patients and healthy controls, independent on the treatment received [39]. Detailed analysis based on the level of habitual physical activity within the age, BMI, and adiposity-matched patients revealed that higher self-reported PA level was related to lower total and LDL-cholesterol in TGCT patients subjected to ≤3 cycles of chemotherapy. However, no PA-related effect was found in those who received >3 chemotherapy cycles.

Presently, there is limited evidence demonstrating casual effects of habitual physical activity or structured exercise training intervention on reducing cardiometabolic risk in TGCT survivors which would consider both the cumulative dose of chemotherapy and time since treatment. In a study on 787 TGCT survivors more than 1 year since treatment with cisplatin-based chemotherapy, higher doses of chemotherapy (3 × BEP vs 4 × BEP) were not linked to higher cardiovascular risk, (FRS: 4.54 vs 4.70, respectively), while vigorous physical activity (*p*= 0.006) and higher education (*p*< 0.001) were associated with lower cardiovascular risk (FRS) [40]. Importantly, at least two previous reports showed that the number of adverse health outcomes observed more than 1 year after receiving cisplatin-based chemotherapy was in 1214 [7] and 952 [41] TGCT survivors negatively correlated with the level of their physical activity.

## Limitations

The study limitations include as follows: (i) our study population (registry) does not include meaningful number of TGCT survivors who received ≤3 chemotherapy cycles, 15–30 years ago. (ii) We only consider time since the last chemotherapy cycle, and not the time-since or age at diagnosis which could have independent effects on cardiometabolic health in TGCT survivors. (iii) Habitual physical activity measure was provided by a single self-reporting event using a validated questionnaire.

## Conclusion

Results from our study show that cardiometabolic health in TGCT survivors is positively linked to the levels of habitual physical activity in patients with stages 1 and 2 of disease, supporting health benefits of daily PA, and advocating for its use in cancer survivors in clinical practice. Patients cured from stage 3 TGCT who received higher cumulative dose of chemotherapy might need structured exercise intervention of higher intensity to induce positive effects on cardiometabolic health.

## Supplementary information

ESM 1 (PDF 952 kb)

## Data Availability

All analyzed and derivative raw data are available on request.
