# Supplementary material for: Habitual physical activity modulates cardiometabolic health in long-term testicular cancer survivors
Source: Support Care Cancer. 2023 Aug 26;31(9):539. doi: 10.1007/s00520-023-08000-1 (PMC10460370; doi:10.1007/s00520-023-08000-1)

Habitual physical activity modulates cardiometabolic health in testicular cancer survivors

Journal of Supportive Care in Cancer

Ali Amiri[1], Patrik Krumpolec[1], Michal Mego[2], Barbara Ukropcová[1,3], Michal Chovanec[2*#], Jozef Ukropec[1*#]

[1.] Department of Metabolic Disease Research & Center of Physical Activity Research, Institute of Experimental Endocrinology, Biomedical Research Center, Slovak Academy of Sciences, Bratislava, Slovakia

[2.] 2nd Department of Oncology, Faculty of Medicine, Comenius University and National Cancer Institute, Bratislava, Slovakia

[3.] Institute of Pathophysiology, Faculty of Medicine, Comenius University, Bratislava, Slovakia

Corresponding authors*:

Jozef Ukropec, PhD, DSc
E-mail: jozef.ukropec@savba.sk
Orcid: https://orcid.org/0000-0001-8401-6621

A/prof. Michal Chovanec, MD, PhD
E mail: michal.chovanec@nou.sk
Orcid: https://orcid.org/0000-0002-5653-2909

Supplementary figure 1. The correlation between Beacke`s habitual physical activity and the 7-day objective habitual physical activity measurements provided by accelerometers

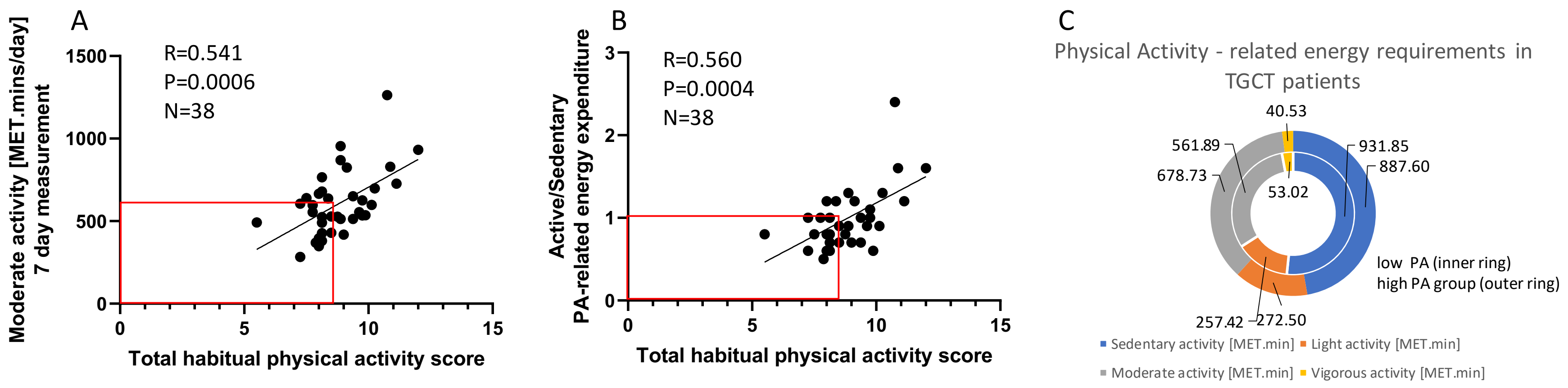

Total habitual physical activity score correlated with the intensity of habitual PA determined by accelerometry (24h moderate intensity PA in MET.min) (A), as well as with the sedentary behavior measure expressed as a ratio of more intensive Physical Activity vs. Sedentary Activity-related daily energy requirements (B). TGCT patients with low physical activity spent more time engaging in sedentary activities and less time engaging in activity with moderate or high intensity, e.g., physical activity requiring >3METs (C). This clearly demonstrates specific differences in volume and intensity of habitual PA between TGCT survivors in low and high PA groups. PA, Physical Activity; MET, Metabolic Equivalent (a multiple of baseline metabolic rate).

Supplementary figure 2. Study flowchart

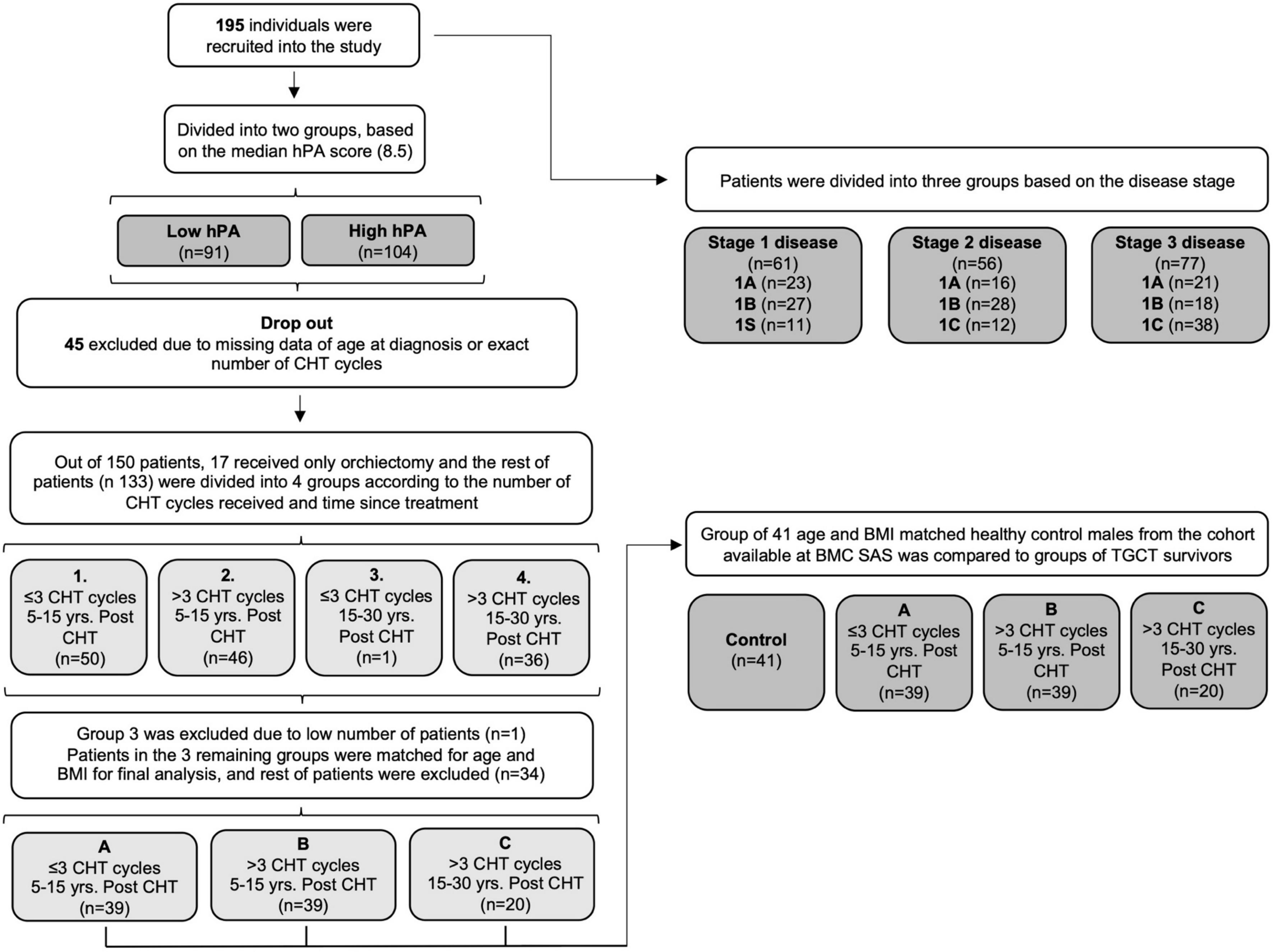

Supplement: Supplementary file 1 — (PDF 952 kb) [file 520_2023_8000_MOESM1_ESM.pdf]
